# Simulations of collisional effects in an inner-shell solid-density Mg X-ray laser

**DOI:** 10.1098/rsta.2022.0218

**Published:** 2023-08-21

**Authors:** Shenyuan Ren, Sam Vinko, Justin S. Wark

**Affiliations:** Department of Physics, Clarendon Laboratory, University of Oxford, Parks Road, Oxford OX1 3PU, UK

**Keywords:** x-ray laser, plasma, free-electron-laser

## Abstract

Inner-shell Kα X-ray lasers have been created by pumping gaseous, solid, and liquid targets with the intense X-ray output of free-electron lasers (FELs). For gaseous targets lasing relies on the creation of K-shell core holes on a time-scale short compared with filling via Auger decay. In the case of solid and liquid density systems, collisional effects will also be important, affecting not only populations but also line-widths, both of which impact the degree of overall gain, and its duration. However, to date, such collisional effects have not been extensively studied. We present here initial simulations using the CCFLY code of inner-shell lasing in solid-density Mg, where we self-consistently treat the effects of the incoming FEL radiation and the atomic kinetics of the Mg system, including radiative, Auger and collisional effects. We find that the combination of collisional population of the lower states of the lasing transitions and broadening of the lines precludes lasing on all but the Kα of the initially cold system. Even assuming instantaneous turning on of the FEL pump, we find the duration of the gain in the solid system to be sub-femtosecond.

This article is part of the theme issue ‘Dynamic and transient processes in warm dense matter’.

## 1. Introduction

The concept of producing short-wavelength lasers via the photoionization of inner-shell electrons was put forward more than half a century ago, when detailed calculations of the requirements for lasing on the K-shell of sodium and copper were performed [1]. Owing to the large power densities and short rise times required for pumping, the practical realization of such ideas took some considerable time to be realized in the X-ray regime [2–4], although lasing in the extreme ultraviolet (XUV) was successfully demonstrated with other schemes based on collisional excitation [5] or recombination [6] in highly ionized plasmas [7,8].

The photoionization approach was first successfully demonstrated in the optical regime (0.325 μm) where laser plasma-produced soft X-rays were used to photoeject a d-shell electron from neutral Cd [9]. A similar concept with the higher Z material Xenon allowed for an extension into the soft X-ray regime at 0.1089 μm [10]. As noted earlier, extension to the keV photon energy regime is much more difficult, owing to the nonlinear scaling of the required pump power with the inverse of the wavelength of the lasing transition. For this reason, successful demonstration of a K-shell X-ray laser [2–4] had to await the development of the high brightness X-ray pump sources provided by X-ray free-electron lasers (FELs) [11], which can create copious K-shell holes leading to population inversion.

The requirement for high pump power is related to the short lifetime of the relevant transition, whereby the lifetime of a K-shell hole created in an isolated neutral atom or ion will normally be dominated by the Auger decay rate. For mid-Z elements, the Auger lifetime is typically in the femtosecond regime. In a low-density system, the Auger rate will be the self-terminating factor, while at higher densities, electron collisional ionization becomes important. As pointed out by Kapteyn [12] in a dense system, the electrons that are ejected into the continuum due to photoionization of the K-shell and the subsequent Auger decay processes will, via collisional processes, produce copious higher ionization stages in their ground state, which comprise the lower laser level of the K-shell transitions, thus potentially curtailing gain. For a system of sufficiently low density, such collisional effects should not be important, and the rate of pumping is determined simply by the requirement to overcome the Auger decay rate. Detailed simulations by Rohringer *et al.* indicated that the X-ray intensities afforded by hard X-ray FEL systems would be capable of achieving gain in a neon gas [13,14]; a prediction that was subsequently verified experimentally by the same lead author a few years later in the first successful demonstration of a K-shell X-ray laser. In that experiment, performed at the Linac Coherent Light Source (LCLS), gaseous neon was irradiated by 0.96 keV X-rays at an intensity of order $2\times 10^{17}\;W\;{\mathrm{cm}}^{-2}$ in pulses between 40 and 80 fs in duration. Lasing was observed on the Kα transition of neutral neon at 0.849 keV [2].

Whilst K-shell lasing in a solid-density system is in principle more difficult owing to the collisional effects mentioned earlier, such have been the advances in FEL technology that a few years later gain was observed on the Kα1 and Kα2 transitions of Cu at 8.04 and 8.02 keV, respectively. In these experiments, performed at the SACLA BL3 facility [15], the FEL output, of 7 fs duration, was focused onto solid Cu targets at irradiances of several times $10^{19}\;W\;{\mathrm{cm}}^{-2}$. The authors also used a two-colour FEL scheme to seed the lasing transition [3]. Further developments have been made in lasing in liquid systems. In experiments at LCLS, stimulated emission has been observed on the Kα1 transition of Mn by the irradiation of two different Mn compounds, ${\text{Mn( II) Cl}}_{2}$ and ${\text{NaMn( VII) O}}_{4}$, in aqueous solutions. In these experiments, the LCLS beam was tuned to an energy of 6.6 keV, with a pulse length estimated to be between 10 and 30 fs at an intensity of order $10^{20}\;W\;{\mathrm{cm}}^{-2}$. Detailed measurements of the lasing output provided estimates of a gain of order $2\times 10^{6}$ over the spontaneous emission [4], with an assumed output pulse duration of order 1 fs. Interestingly, the photon energy of the Kα1 emission was found to be slightly different for the two solutions, indicating that the effect of the chemical environment was preserved during the lasing process, hinting that significant gain existed before any outer-shell ionization had occurred.

The work cited earlier demonstrates the interest in generating K-shell X-ray lasers using FELs as the pump, and it is in this context that we present here our initial simulations of K-shell lasing in both low-density and solid-density Mg. This study is in part motivated by the observation that most simulations or calculations to date of FEL pumped K-shell lasing have been for low-density systems [3,13,14,16], rather than explicitly taking collisional effects into account.

We choose Mg for a number of reasons. Firstly, as a relatively low-Z material the atomic physics is less complex, reducing computational cost. Secondly, there have been several experimental studies of the spontaneous emission spectra emitted by solid-density Mg targets illuminated by an FEL, and comparison of those spectra with previous simulations with the CCFLY code used here provides confidence that the overall evolution of the charge state distribution in the solid-density plasma is relatively well understood [17–19]. Lastly, with an atomic number of 12, it does not differ too much from that of neon (Z=10), allowing at least some degree of comparison of results (at least in the low-density case) with those of Rohringer, although the details of the calculations differ in several respects, which we outline in the following sections.

## 2. Simulations

Simulations were performed using the collisional-radiative code CCFLY, which itself is an updated (written in C++) version of the SCFLY code described briefly elsewhere [20], with both these codes being substantially revised versions of the widely available FLYCHK suite [21,22]. CCFLY is a non-local-thermodynamic-equilibrium (non-LTE) code in that the emitted radiation and ionic ground and excited-state populations are evolved in time, rather than assuming thermodynamic equilibrium. Note, however, that in the version of the code used here, electrons in the continuum are assumed to obey classical statistics, and to instantaneously thermalize to a temperature dictated by their overall energy content. Within that caveat, CCFLY is specifically tailored for X-ray laser problems in that it provides a self-consistent electron temperature calculation derived by the energy balance between the absorbed FEL radiation, internal energies of the electrons and ions in the system, and the emitted radiation. Whilst energy can be transferred to the atoms resulting in ionization and excitation, in contrast with the electrons we make the assumption that on the time scale of typical FEL pulses, no kinetic energy is given to the ions, such that they are assumed to remain at room temperature throughout the calculation. We consider such an assumption to be valid, given calculations that indicate that the time scale for electron-ion equilibration, in terms of their temperatures, is several picoseconds [23–25].

CCFLY treats the atomic physics in terms of superconfigurations, which denote the number of electrons with a specific principal quantum number, i.e. the number of electrons within the K, L or M shells. In solid Mg, which is metallic, the M shell electrons are already effectively ionized, giving the ground state superconfiguration (KLM) of (280). This is taken into account by the introduction of a continuum lowering (ionization potential depression (IPD)) routine within the code. Various models for such IPD have been put forward, with studies of the K-shell emission spectra from Al and Mg, indicating that a modified version of the Ecker–Kroll IPD model [26] provides results consistent with experiments under these conditions, at least for the first few charge states [17,27], although the exact model used does not affect the results presented here in any significant way.

The X-ray laser radiation field used in the calculations is assumed to be constant in time and Gaussian in frequency, with a fractional bandwidth of 0.4% and a photon energy of 2000 eV. The intensity of the X-ray pulse is $3\times 10^{17}W\;{\mathrm{cm}}^{-2}$. In contrast to the work of Rohringer *et al.* [13,14], we make no attempt in these initial studies to model the effect of the rapid temporal modulations in FEL intensity caused by the SASE (self-amplified spontaneous emission) spikes, as our overarching aim is the more modest goal of elucidating the principal differences between the low- and high-density cases.

The ion density for solid Mg is $4.3\times 10^{22}\;{\mathrm{cm}}^{-3}$. For comparisons with previous work, we also perform calculations at a density 4 orders of magnitude lower, at $4.3\times 10^{18}\;{\mathrm{cm}}^{-3}$, which is both sufficiently small so as to make collisional effects negligible, and is similar to the values previously used for the neon gas targets. For ease of comparison with the solid-density case, we also assume that the starting superconfiguration of the low-density case is (280), i.e. that it is doubly ionized before the onset of the FEL pulse (one could envisage the production of such a low-density target by optical laser ablation of an Mg foil).

The simulations provide the populations of the various superconfigurations as a function of time, which in turn allow us to calculate the gain (or otherwise) of the relevant transitions. The gain cross-section for a particular transition, $\sigma_{\text{stim}}$, is given by

2.1 $$\sigma_{\text{stim}}=\frac{2\pi c^{2}A}{\omega^{2}\Delta \omega },$$

where the A is the spontaneous emission rate in $s^{-1}$ and Δω is the width of the transition. For the low-density case, this width will be dominated by the Auger lifetime (which, e.g. for the transition from the (180) to (270) superconfigurations results in a width of order 0.4 eV). However, at solid densities, the line width can increase due to collisional ionization. We take this into account in a very simple model where we assume an additional line width, which is a function of the sum of the inverse of the relevant bound-bound and bound-free collisional rates for the upper and lower levels. As we shall see below, the precise form of this additional width does not play a significant role in affecting the overall gain in these calculations. The gain per atom is given by

2.2 $$g(t)=\left(N_{u}(t)-N_{l}(t)*\frac{g_{u}}{g_{l}}\right)\frac{\sigma_{\text{stim}}(t)}{N_{i}},$$

where $N_{u}(t)$ and $N_{l}(t)$ are the populations of the upper and lower state at time t; $g_{u}$ and $g_{l}$ are the respective degeneracies; and $N_{i}$ is the total ion density. Note the gain cross-section is now considered to be time dependent owing to the effect of collisions on the linewidth.

## 3. Results

**Figure 1.**
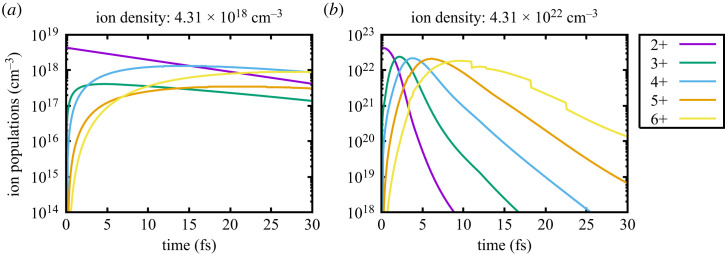
The charge state distribution as a function of time for low (*a*) and solid (*b*) density Mg.

Before turning our attention to lasing itself, in figure 1, we plot the populations of the ground state ions (i.e. those with two electrons in the K shell, and varying numbers of L-shell electrons) as a function of time for both the low- and high-density cases. Note all possible charge states are included in the simulations, but only the relevant ones are shown here. It can be immediately seen that in the low-density case, the charge states that predominate have an even number of L-shell electrons, whereas in the solid-density case, as time proceeds, all of the successive charge states are produced. For example, at a time of 30 fs in the low-density case, charge states 4+ and 6+ have populations several times greater than those of 3+ and 5+. This alternation in the populations of the charge states in the low-density case was demonstrated in one of the first experiments ever to be performed at LCLS, where the charge states produced when neon gas was irradiated by the FEL pulse were measured [28]. The results agreed well with simulations, including those undertaken using a previous version of the code used here [29]. This phenomenon is due to the fact that (assuming the FEL X-ray energy chosen lies above the K-shell photoionization energy for all of the charge states to be considered), after each K-shell photoionization event, creating a K-shell hole, the dominant decay mechanism will be via the Auger effect, resulting in the filling of the K-shell hole by an L electron, and the ejection of another L electron into the continuum: thus, the absorption of the FEL photon results in double ionization. In contrast, for the solid-density case, the high electron density can cause collisional ionization of the L-shell, resulting in all of the ground state ions being produced, as can readily be seen in figure 1 (note the small kinks in the populations of charge state 6+ in the solid case are due to the prediction by the IPD model used—Stewart-Pyatt—of rebinding of higher atomic shells. As we shall see, lasing will not occur on these higher charge states, and as stated in §1, our overall conclusions are independent of IPD model chosen). This lack of alternation in the charge states produced in the solid-density case was commented upon in work reporting the first observation of X-ray spectra from solid targets irradiated by the focused output of the LCLS [30]. Indeed, as we shall see, it is this very effect that reduces the gain per atom in the solid-density case. Photoionization of the (280) atom creates the (180) superconfiguration, which is the upper state of the K-shell lasing transition, which has a lower state of (270). As the relative number of (270) ions is much greater in the solid-density case, for the reasons given earlier, the creation of conditions conducive to lasing is much more difficult.

**Figure 2.**
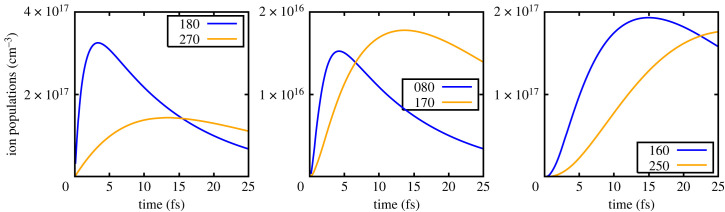
Low density: ion populations of the lower and upper states as a function of time.

**Figure 3.**
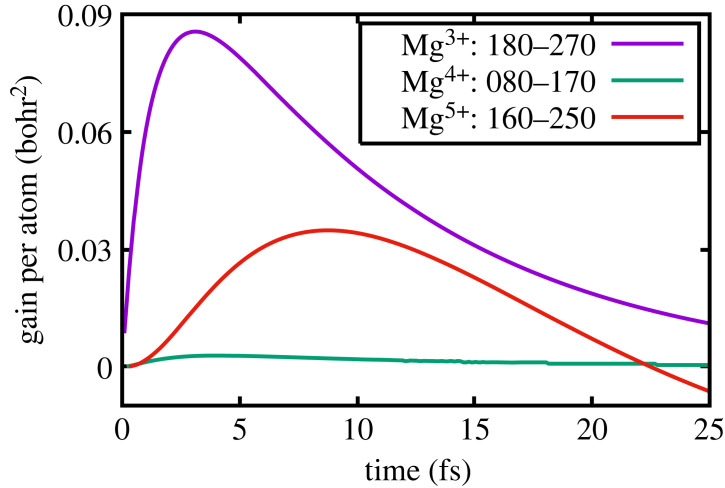
Low density: gain per atom of the transitions.

**Figure 4.**
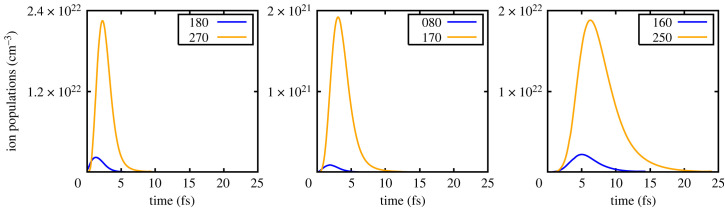
Solid density: ion populations of the lower and upper states as a function of time.

**Figure 5.**
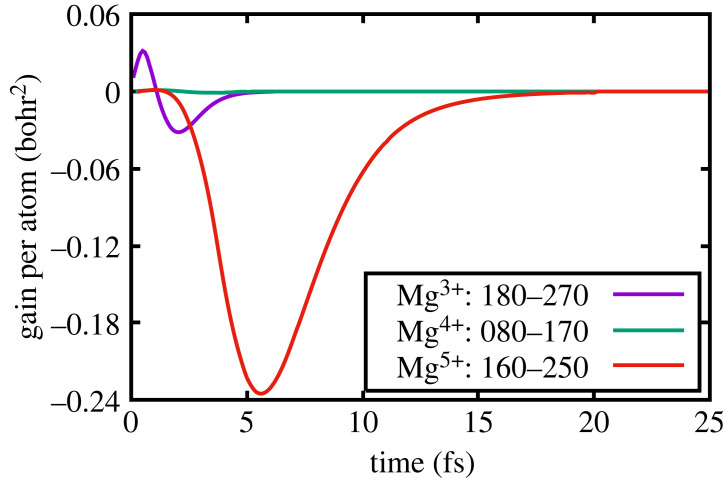
Solid density: gain per atom of the transitions.

To illustrate this further, we show in figure 2 the populations as a function of time, in the low-density case, of (180) and (270), (080) and (170) (given that it is possible to lase due to the creation of double core holes) and finally, (160) and (250). The corresponding gains per atom are shown in figure 3 in units of (bohr)${\;}^{2}$. It can be seen that for Mg3+, lasing on the (180)–(270) transition, the gain per atom peaks after approximately 3-fsec at a value of $0.085\;{\mathrm{bohr}}^{2}$. This gain value is very similar to that found by Rohringer *et al.* in their numerical studies of lasing in neon, and given the atomic numbers are close, and the pumping conditions not too dissimilar, such agreement is encouraging. Furthermore, these values are also consistent with the gain values found in other simulations of the neon system where at atomic densities of $2\times 10^{19}\;{\mathrm{cm}}^{-3}$ gains of order $60\;{\mathrm{cm}}^{-1}$ were predicted [16] (for this atomic density, our prediction for Mg would correspond to of order $40\;{\mathrm{cm}}^{-1}$). Note, however, that the gain values that we quote are for a superconfiguration, i.e. to say the gains for transitions between particular configurations have been summed (e.g. our gain for (180)–(270) is effectively the sum of gains for Kα1 and Kα2).

We note that, as found by Rohringer, the system can also lase on the Kα transitions of higher charge states. Owing to the charge states with significant populations alternating due to Auger decay, as described earlier, the next Kα transition with significant gain is that between the (160) and (250) superconfigurations, where the peak gain is slightly less than half that of the (180)–(270) transition. We also note that owing to the high photoionization rate of the K-shell, a small gain per atom is predicted on the transition with an upper state containing a double core hole: (080)–(170), but at a peak value of $0.0028\;{\left(\text{bohr}\right)}^{2}$, at these densities, this would only translate to a gain of order $1.3\;{\mathrm{cm}}^{-1}$, which, whilst it could prove difficult to experimentally verify, may still be measurable.

**Figure 6.**
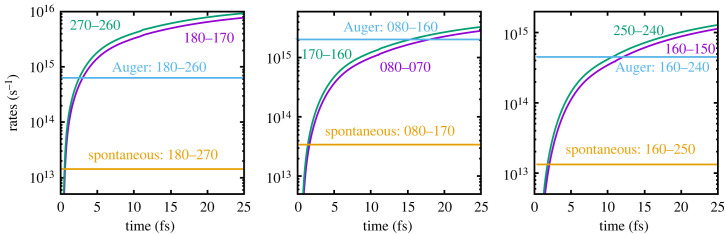
Solid density: the spontaneous, Auger and collisional rates as a function of time for transitions between various superconfigurations. (Note, for low density, the Auger and spontaneous rates remain unchanged, but the collisional rates become negligible compared with them.)

**Figure 7.**
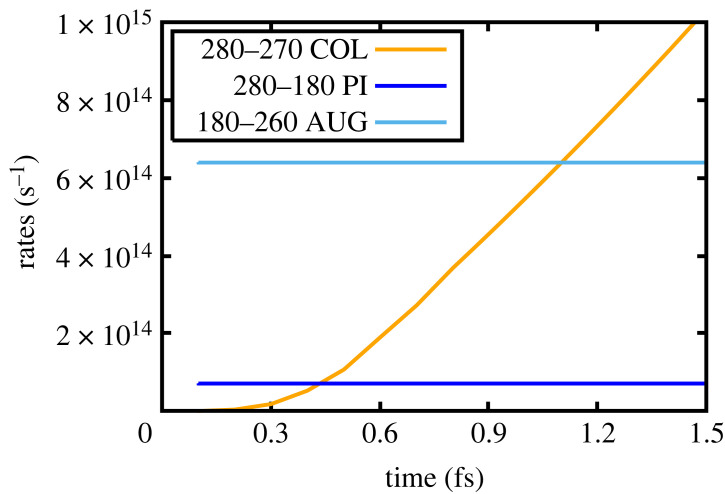
Solid density: collisional rate of 280–270, the photoionization rate of (280)–(170) due to the FEL and the Auger rate (180)–(260) as a function of time.

This situation should be contrasted with that of the solid-density case, where the populations of the relevant superconfigurations as a function of time are shown in figure 4. Whilst here K-shell photoionization starts to produce the upper state (180), as noted earlier, the electrons that are ejected in this process can rapidly ionize the L-shell of (280), producing copious quantities of ions in the (270) superconfiguration, which is the lower state of the first lasing transition. Thus, we can see in figure 4 that on a time scale of order a femtosecond, the ground state population exceeds that of the upper state to such a degree that even taking into account the different degeneracies the gain quickly goes negative, as shown in figure 5. Indeed, the effect of collisions is such that for all of the subsequent charge states, no gain whatsoever is observed. For the first ion stage (i.e. the initial ion in the cold metal having had 1 K-shell electron photoionized), the gain per atom peaks at $0.024\;{\mathrm{bohr}}^{2}$, more than a factor of 3 lower than in the low-density case, and this is even with us assuming an instantaneous turning on of the FEL. These results clearly demonstrate the more stringent requirements for lasing in the solid-density case. Indeed, the very short duration of the gain, even in the situation where within the simulation, we have assumed instantaneous turn-on of the X-ray pulse, may provide some explanation as to why, for a given total energy in the X-ray pump, there are far greater variations in the gain seen experimentally in the liquid case than in the experiments with neon gas: for example compare figure 3 of reference [2] with figure 1*c* of reference [4].

Further insight into the aforementioned processes can be gleaned from plots showing the relevant rates as a function of time for the various transitions pertinent to the lasing processes; these are plotted in figures 6 and 7. From figure 6, we see that the Auger rate would dominate in the absence of collisions and is nearly two orders of magnitude greater than the spontaneous rate (with the ratio of the spontaneous to Auger+spontaneous rate being the fluorescence yield). We note that for all three transitions shown the collisional ionization rates rapidly dominate over the Auger rate. In particular, and as shown in figure 7, we see that the collisional ionization rate that produces the lower state of the first lasing transition, i.e. the superconfiguration (270), exceeds the Auger rate of (180)-(260) on a time scale of order a femtosecond.

**Figure 8.**
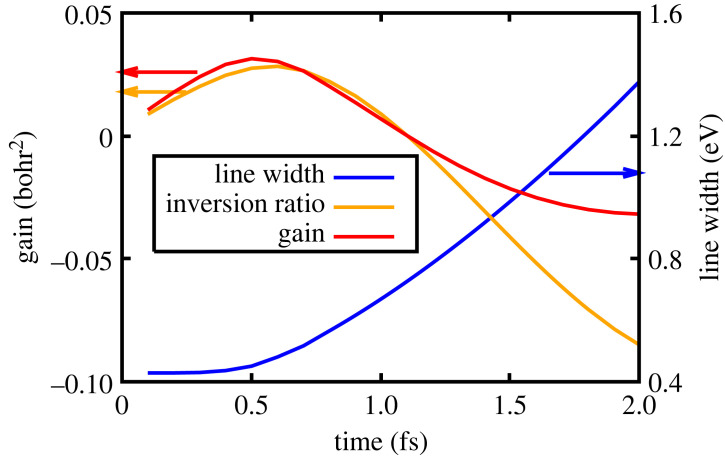
Solid density: the gain (left axis in units of ${\mathrm{bohr}}^{2}$), inversion ratio (left axis in dimensionless units) and line width (right axis in units of eV) as a function of time for the (180)–(270) transition.

Thus far, we have considered the curtailing of the gain in the solid-density case as being due to collisional effects rapidly populating the ground state of the transition. However, as noted earlier, such collisions also reduced the gain cross-section by increasing the line width of the transition. The relative importance of these two effects can be seen in figure 8, where we show the gain per atom on the (180)–(270) transition, the linewidth and the effective population inversion (i.e. $\left[N_{u}(t)-N_{l}(t)*g_{u}/g_{l}]/N_{i}\right]$) as a function of time. It can be seen that the peak gain occurs very slightly earlier (about 0.1 fs) than the peak in the inversion ratio, and that this is due to the increase in the line width. However, the curtailment of the gain is clearly dominated by the changes in the populations, that is to say the inversion ratio itself, with the increasing width of the lasing transition only playing a minor role.

A further difference, however, between the low- and high-density cases is the duration of positive gain: 11 fs full width half maximum (FWHM) in the low-density case for the (180)–(270) transition, yet just 0.65 fs (FWHM) in the solid-density case. We see that whilst the peak gain per atom is reduced owing to the collisional effects, this very same mechanism also results in a reduced pulse length of the laser, which may well have practical advantages if such systems are subsequently to be used in further applications, especially as the pulse length appears to be approaching that of the standard unit of atomic time, $a_{0}/(\alpha c)=24.3$ as.

## 4. Discussion

There are several directions which appear fruitful for further study. Given the extremely short time scales over which gain in the solid state system exists owing to collisional effects, it is evident that any improvements in understanding of the relevant rates could well influence the degree and duration of gain predicted. It should be noted that the rates used in this study have been the those used in previous versions of the SCFLY code, whereas recent experimental work has shown that these rates may well be an underestimate [19,31]. Furthermore, here the assumption has also been made that the distribution function of the electrons in the continuum is at all times Maxwellian—i.e. we have both ignored the fact that the cold metal will have a Fermi-Dirac distribution of the electrons, and we have further assumed that thermalization of the electrons is instantaneous.

**Figure 9.**
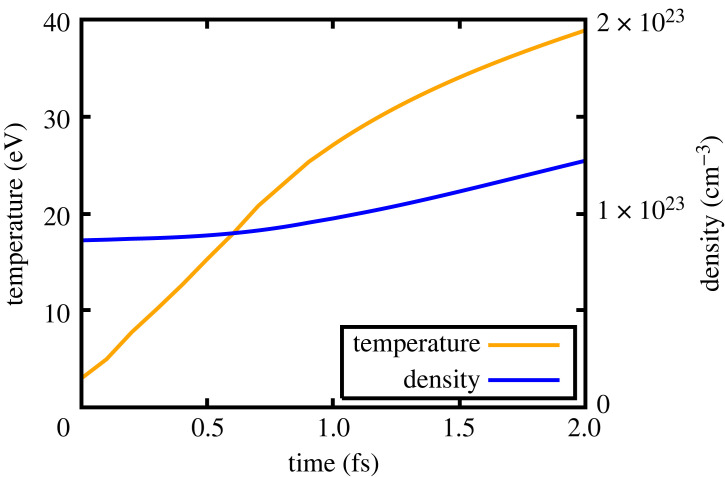
Solid density: temperature and electron number density as a function of time.

Both of the aforementioned assumptions warrant scrutiny. If, for the sake of argument, we do assume instantaneous thermalization, the fact that the current version of the code does not take into account the Fermi degeneracy of the initial electrons is unlikely to be significant because the electron temperature exceeds the Fermi temperature on exceedingly short time scales. Figure 9 shows the calculated electron temperature and density as a function of time in the solid-density case. The electron temperature rise exceeds the Fermi energy of Mg (4.4 eV), justifying classical statistics in less than 0.2 fs.

However, such is the brevity of the gain in the solid system that it may well be that the assumption of instantaneous thermalization of the electrons needs to be reconsidered, given that electrons are ejected into the continuum via photoionization and Auger decay at specific energies. A more recent version of the code used here has been developed that, with considerable increased computational cost, keeps track of the evolving non-thermal electron distribution function and alters the collisional rates used accordingly [32]. Further studies incorporating this capability would clearly be beneficial. Extending studies to materials of higher atomic number (again at potentially greater computation cost) would also be of interest. We also note that owing to the extremely short duration of the predicted gain in the solid-density case, we might very well expect considerable differences in gain if we relax the assumption of a top-hat FEL pulse-i.e. to say the statistics of the SASE spikes, considered by Rohringer in the low density case [13,14], might well play an equal if not even more important role in the statistics of the Kα lasing output in the solid-density case. Finally, the current version of CCFLY treats the system in terms of superconfigurations. Whilst tracking the populations of the myriad of possible configurations as a function of time would be computationally prohibitive in many simulations of FEL-matter interactions, in the case of K-shell lasing in solid or liquid density systems, the work presented here indicates that only the first one or two charge states will be of any real significance. Thus, a more detailed treatment of the atomic physics, including collisional effects, of these few states may well be feasible in future studies.

## Data Availability

This article has no additional data.

## Appendix A

From the results presented in this article, it is clear that electron collisional ionization plays an important role in determining the lifetime of states that may show gain. We expand on the details of our modelling approach here.

The electron collisional ionization rate S of some ion is given by the average of the collisional ionization cross section σ over the distribution of available electron velocities v, i.e. S=⟨σv⟩. In our modelling, we assume a Maxwellian distribution for the free-electron population, and that this distribution thermalizes instantaneously at each time step of the collisional-radiative calculation. Then, we can write the rate explicitly as follows:

A 1 $$S=n_{e}\sqrt{\frac{8}{\pi m_{e}}}{\left(k_{B}T\right)}^{-3/2}\int_{0}^{\infty }\varepsilon \sigma (\varepsilon )\;e^{-\varepsilon /k_{B}T}\;d\varepsilon .$$

Here, $n_{e}$ is the density, T the temperature and $m_{e}$ the mass of the free electrons. The integral is taken over the electron energy. There are many possible choices for the collisional ionization cross section for dense systems (e.g. see van den Berg *et al.* [33] and references therein for a more detailed discussion). In this work, we have used the simple empirical Lotz model [34], where the energy-dependent collisional cross-section is given by the following expression:

A 2 $$\sigma (\varepsilon )\approx \sum_{i}a_{i}q_{i}\frac{\ln(\varepsilon /P_{i})}{\varepsilon P_{i}},$$

where the sum is over all atomic sub-shells. The $a_{i}$ terms are fitting constants [35], $q_{i}$ denotes the population of the sub-shell and $P_{i}$ is the ionization energy of a particular sub-shell. The effect of continuum lowering can be included in the model by an appropriate re-scaling of the ionization energy. We have verified that the cross-sections calculated from this simple model are comparable to more advanced calculations based on the binary encounter, distorted wave or scaled hydrogenic models [33].
